# Spatio-temporal mapping of breast and prostate cancers in South Iran from 2014 to 2017

**DOI:** 10.1186/s12885-020-07674-8

**Published:** 2020-11-30

**Authors:** Mahdieh Montazeri, Benyamin Hoseini, Neda Firouraghi, Fatemeh Kiani, Hosein Raouf-Mobini, Adele Biabangard, Ali Dadashi, Vahideh Zolfaghari, Leila Ahmadian, Saeid Eslami, Robert Bergquist, Nasser Bagheri, Behzad Kiani

**Affiliations:** 1 Medical Informatics Research Center, Institute for Futures Studies in Health, Kerman University of Medical Sciences, Kerman, Iran; 2 Health Information Technology, School of Management and Medical Information Science, Kerman University of Medical Sciences, Kerman, Iran; 3 Pharmaceutical Research Center, Mashhad University of Medical Sciences, Mashhad, Iran; 4 Department of Health Information Technology, Neyshabur University of Medical Sciences, Neyshabur, Iran; 5 Department of Medical Informatics, School of Medicine, Mashhad University of Medical Sciences, Mashhad, Iran; 6 Department of Health Information Technology, Faculty of Paramedicine, Mashhad University of Medical Science, Mashhad, Iran; 7 Medical Records Department, Vali-e-asr Hospital, Zanjan University of Medical Sciences, Zanjan, Iran; 8 Department of Medical Educational Technology, Faculty of Medicine, Mashhad University of Medical Sciences, Mashhad, Iran; 9 Ingerod, SE-454 94 Brastad, Sweden. Formerly UNICEF/UNDP/World Bank/WHO Special Programme for Research and Training in Tropical Diseases (TDR), World Health Organization, Geneva, Switzerland; 10 Visualisation and Decision Analytics (VIDEA) Lab, Centre for Mental Health Research, Research School of Population Health, College of Health and Medicine, The Australian National University, Canberra, Australia

**Keywords:** Spatial analyses, Cluster analyses, Breast Cancer, Prostate Cancer, Spatio-temporal, Geographical information systems, Iran

## Abstract

**Background:**

The most common gender-specific malignancies are cancers of the breast and the prostate. In developing countries, cancer screening of all at risk is impractical because of healthcare resource limitations. Thus, determining high-risk areas might be an important first screening step. This study explores incidence patterns of potential high-risk clusters of breast and prostate cancers in southern Iran.

**Methods:**

This cross-sectional study was conducted in the province of Kerman, South Iran. Patient data were aggregated at the county and district levels calculating the incidence rate per 100,000 people both for cancers of the breast and the prostate. We used the natural-break classification with five classes to produce descriptive maps. A spatial clustering analysis (Anselin Local Moran’s *I*) was used to identify potential clusters and outliers in the pattern of these cancers from 2014 to 2017.

**Results:**

There were 1350 breast cancer patients (including, 42 male cases) and 478 prostate cancer patients in the province of Kerman, Iran during the study period. After 45 years of age, the number of men with diagnosed prostate cancer increased similarly to that of breast cancer for women after 25 years of age. The age-standardised incidence rate of breast cancer for women showed an increase from 29.93 to 32.27 cases per 100,000 people and that of prostate cancer from 13.93 to 15.47 cases per 100,000 during 2014–2017. Cluster analysis at the county level identified high-high clusters of breast cancer in the north-western part of the province for all years studied, but the analysis at the district level showed high-high clusters for only two of the years. With regard to prostate cancer, cluster analysis at the county and district levels identified high-high clusters in this area of the province for two of the study years.

**Conclusions:**

North-western Kerman had a significantly higher incidence rate of both breast and prostate cancer than the average, which should help in designing tailored screening and surveillance systems. Furthermore, this study generates new hypotheses regarding the potential relationship between increased incidence of cancers in certain geographical areas and environmental risk factors.

**Supplementary Information:**

The online version contains supplementary material available at 10.1186/s12885-020-07674-8.

## Background

Cancers are the second leading cause of death worldwide [1], which can partly be explained by the fact that the world’s population is ageing [2]. Furthermore, human exposure to multiple risk factors has increased the cancer burden worldwide [3]. Despite advances in timely diagnosis and medical treatment of neoplasms in recent years, malignancies in middle to low-income countries are expected to almost double by 2030 compared to high-income nations [4]. If cancer is diagnosed promptly, cures can sometimes be found and life prolonged leading to considerably lower disease burdens [5, 6]. However, health systems, particularly in developing countries, are not capable of screening all people to identify patients in the early stages of the disease. Identifying high-risk geographical areas could help decreasing the cost of screening, finding the people at risk and implementing more efficient diagnostic strategies [7]. Investigating high-risk areas should also provide valuable knowledge to scientists about the aetiology of some malignancies [3].

Cancer of the breast and the prostate are the two most common, gender-specific malignancies worldwide [8]. Furthermore, these neoplasms cause high numbers of disability-adjusted life years (DALYs) [9]. Risk factors for these two diseases are diverse and interrelated, as they include genetic [10], social-economic [11] as well as lifestyle and environmental factors [12]. Further, there are interactions between these risk factors, particularly with those involving the environment [13, 14], whose spatial variation may lead to heterogeneity in the pattern of cancers in a given geographic catchment area. Studies by Wang et al. [15, 16] found a significant spatial variation of prostate cancer incidence and prostate cancer-specific mortality in Pennsylvania, USA. They evaluated potential effects of individual and county-level risk factors and found that spatial variations in prostate cancer-specific mortality rates existed in Pennsylvania with a particularly high risk in the Pen State catchment area. County-level health and environmental factors might contribute to spatial heterogeneity in prostate cancer-specific mortality as shown by Olfatifar et al. [17], who examined spatial clustering of breast cancer at the provincial level in Iran between 2004 and 2010. Their results highlight that the breast tumour incidence varied across the provinces [17]. At the same time, Rohani-Rasaf et al. [18] detected some high-risk regions in Tehran, the capital of Iran, both for cancers of the breast and the prostate. Most studies in Iran have applied spatial analyses at a very coarse level (province scale) and the results are therefore not as useful as a finer scale quite naturally.

Geographical information systems (GIS) constitute a set of useful tools for the identification of high-risk areas of cancer occurrence as well as investigation of the environmental effects on cancer incidence [19–21]. GIS approaches combine spatial and non-spatial data producing geodatabases that make it possible to perform spatial analyses using this data structure [22, 23]. For example, spatial autocorrelation is a method of exploratory data analysis which allows detecting spatial data dependence [24]. There are two kinds of spatial autocorrelation methods: global and local statistics. Global methods are more sensitive to departures from the null hypothesis, which examine whether data, here patients, are randomly distributed or if there is a spatial pattern. They can identify spatial structures in the pattern of cancer incidence but do not determine where the clusters are. Local cluster statistics, on the other hand, can quantify spatial autocorrelation and clustering, but only in limited areas. These methods may find restricted areas characterized as high-high (HH), high-low (HL), low-low (LL) or low-high (LH) risk of incidence within a region. HH and LL are defined as target areas surrounded by areas with similar incidence rates, while for HL and LH, the target areas are surrounded by areas with dissimilar cancer incidence rates. In other words, HH and LL indicate clusters, while HL and LH point to outliers [25]. This study aimed to identify the spatial patterns of cancer of the breast and the prostate and to investigate the potential clustering in gender-specific patterns of these cancers in southern Iran between 2014 and 2017.

## Method

### Study area and time

**Fig. 1:**
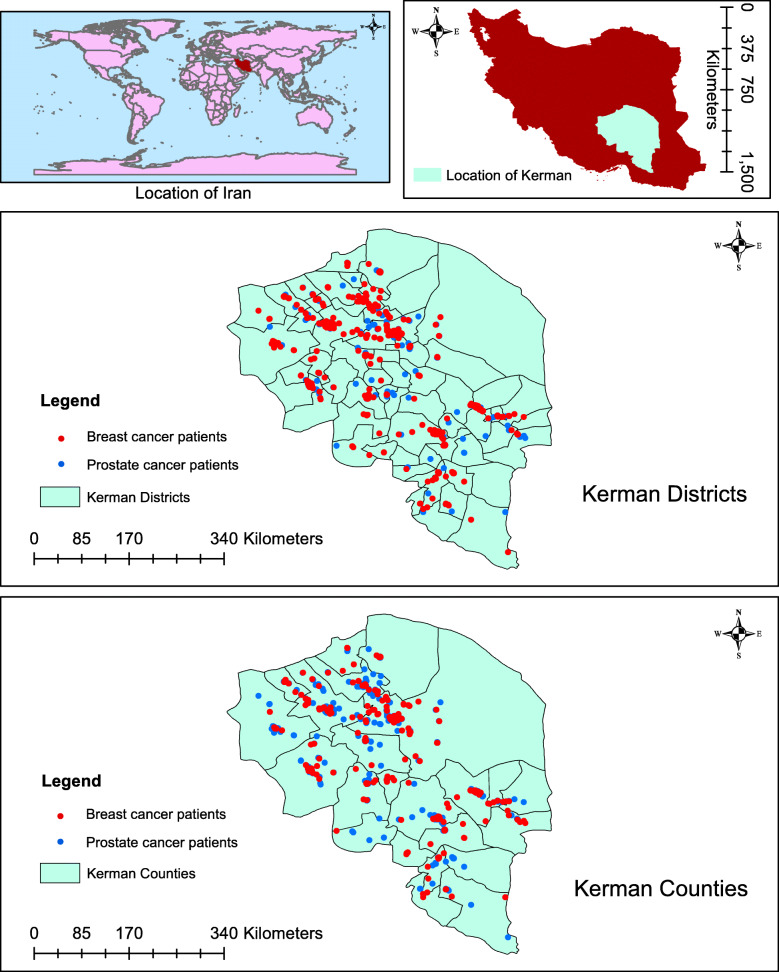
Map of Kerman counties and districts including the distribution of cancer (prostate and breast) patients during 2014–2017. The figure was created by authors

This study was conducted in the province of Kerman, located in southern Iran (Fig. 1). The first administrative level of Iran subdivisions is the province, each of which is further divided into counties that are in turn divided into districts. Our study area contained the 22 counties and 58 districts of Kerman Province, which covers an area of 183,285 km^2^ and has, according to the National Census of 2015, a population of 3,164,718 people [26]. The study covered the time span of March 2014–March 2017.

### Data sources

Data were obtained from two different sources with three different spatial scales (individual, county and district). The individual patient data (supplementary files 1–2) were obtained through the population-based cancer registry of Kerman. They were geocoded and aggregated to both county and district. The digital maps (county and district) were obtained through the mapping organisation of the country.

We used a crude incidence per 100,000 people and the age-standardised rate (ASR) per 100,000 people for the descriptive statistics.

### Inclusion and exclusion criteria

We included all residents of Kerman diagnosed with either cancer of the breast or the prostate. Individuals who had come to the province for cancer treatment but lived outside of the province were excluded.

### Spatial analyses

For the thematic maps, we used the natural-break classification with five classes. This approach is a data-clustering method designed to determine the best arrangement of values into different classes. This is done by seeking to minimise each class’s average deviation from the class mean, while maximising each class’s deviation from the means of the other groups. In other words, the method seeks to reduce the variance within classes and maximise the variance between them [27]. For spatial visualisation, the crude incidence per 100,000 people and the ASR per 100,000 people were used.

### Spatial Cluster Analysis

Incidence rates of the two target cancers were calculated using total population and number of cases in each county and district of the province. The Local Moran’s *I* statistic [28] was performed to quantify spatial autocorrelation of cancers frequency at county and district level. This test calculates a *z*-score and *p*-value to determine whether the apparent similarity (spatial clustering of either high or low values) or dissimilarity (presence of spatial outliers) is more pronounced than one would expect in a random distribution. The null hypothesis states that the cancers are randomly distributed across the study area. A high positive z-score for a feature indicates that the surrounding features have similar values (either high values or low values). However, a low negative z-score for a feature indicates a statistically significant spatial data outlier [28]. We used a 95% Confidence Level (CL), and all clusters and outliers found in this study were significant at this CL.

We used ArcGIS, v. 10.5 (ESRI, Redlands, CA, USA) and GeoDa (https://spatial.uchicago.edu/geoda) for spatial analyses and Microsoft Excel 2016 for the descriptive analyses.

## Results

There were 1350 breast cancer patients (including, 42 male cases) and 478 prostate cancer patients in the province of Kerman, Iran during the period March 2014–March 2017. Table 1 shows the crude incidence per 100,000 people and ASR per 100,000 people of these cancers. The ASR of both breast and prostate cancers increased by 29.93 to 32.27 and by 13.93 to 15.47 from 2014 to 2017, respectively.

**Table 1:** Breast and prostate cancer incidence per 100,000 people during different time periods in the province of Kerman, Iran

Period	2014–2015			2015–2016			2016–2017		
Cancer:	Number	Crude rate	ASR^a^	Number	Crude rate	ASR^a^	Number	Crude rate	ASR^a^
Female Breast	424	26.64	29.93	425	28.71	32.49	459	29.73	32.27
Male Breast	14	0.90	1.52	20	1.02	1.22	8	0.37	0.42
Prostate	151	9.82	13.93	128	8.42	11.29	199	12.36	15.47

^a^Age-standardised rate

**Fig. 2:**
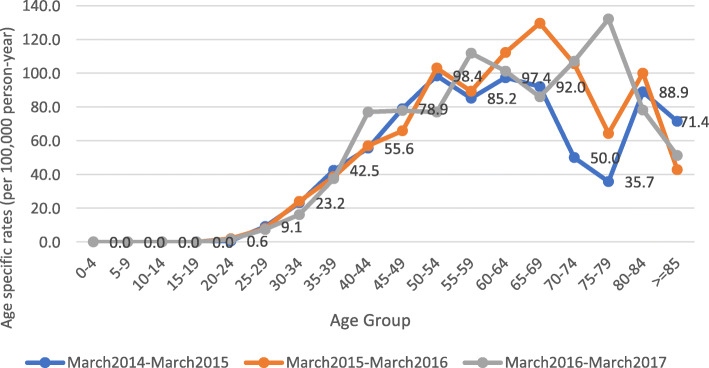
Age distribution of breast cancer patients in the province of Kerman, Iran

As shown in Fig. 2, the number of women (after 25 years of age) who developed breast cancer increased rapidly. Furthermore, the highest incidence occurred in the 50–54 age group in the 2014–2015 period, in the 65–69 age group in the 2015–2016 period and in the 75–79 age group in the 2016–2017 period.

**Fig. 3:**
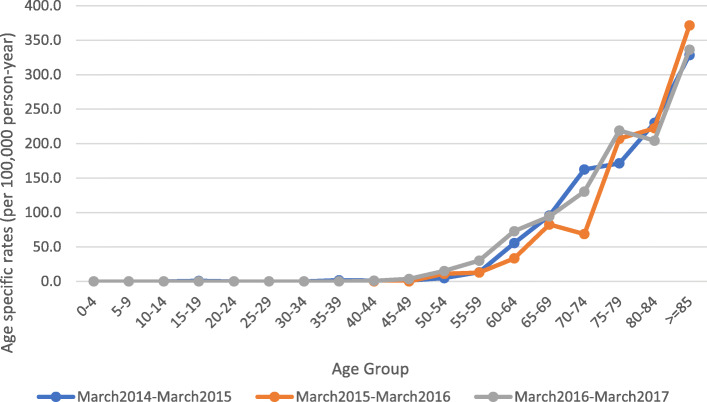
Age distribution of prostate cancer patients in the province of Kerman, Iran

After 45 years of age, the number of men with diagnosed prostate cancer increased similarly to that of breast cancer for women after 25 years of age. In contrast, however, the ascent of this cancer in relation to seniority was considerable in all years from 2014 to 2017 (Fig. 3).

**Fig. 4:**
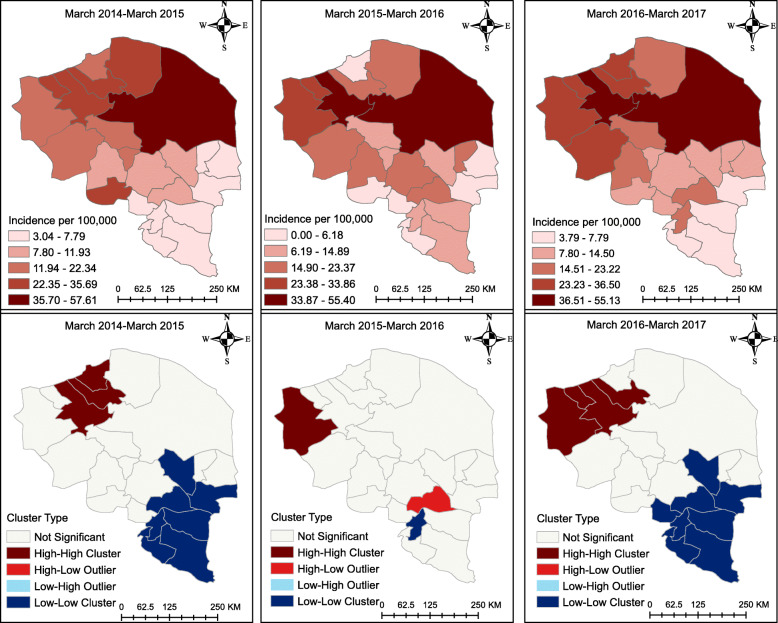
Breast cancer incidence map at the county level in the province of Kerman, Iran. The figure was created by authors

The descriptive maps of Fig. 4 reveal that the breast cancer incidence was highest in the north-eastern part of the province from 2014 to 2017. However, as the cluster maps show, there were HH clusters of breast cancer in the north-western part of the province from 2014 to 2017; furthermore, there was a LL cluster in the South-East in 2014–2015 and in 2016–2017, but not in the time between these periods. All clusters and outliers were statistically significant (*p* < 0.05).

**Fig. 5:**
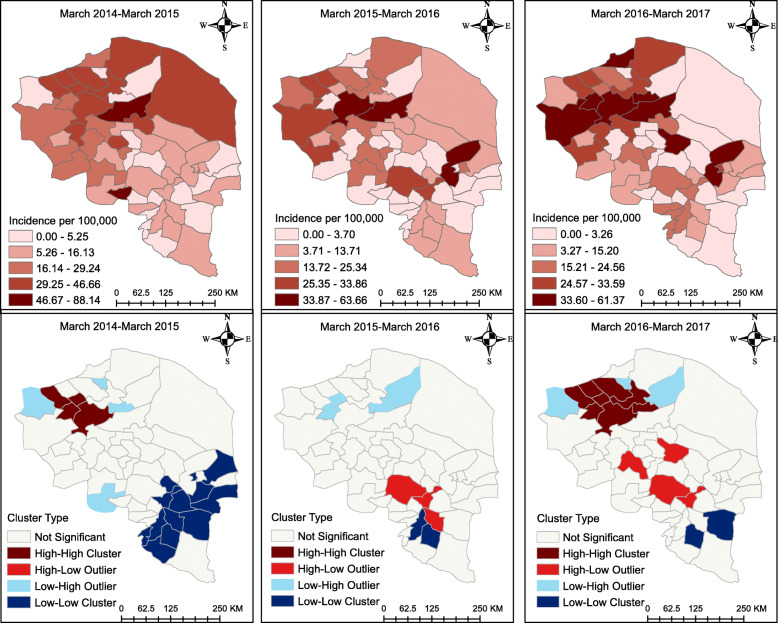
Breast cancer incidence map at the district level in the province of Kerman, Iran. The figure was created by authors

The descriptive maps of Fig. 5 reveal that the breast cancer incidence was highest in the North stretching towards the centre of the province. However, as the cluster maps show, there were HH clusters of breast cancer in the north-western part of the province from 2014 to 2015 and 2016 to 2017; furthermore, there were LL clusters in the north-eastern part of the province from 2014 to 2015 and from 2016 to 2017. All clusters and outliers in the figure were statistically significant (*p* < 0.05).

**Fig. 6:**
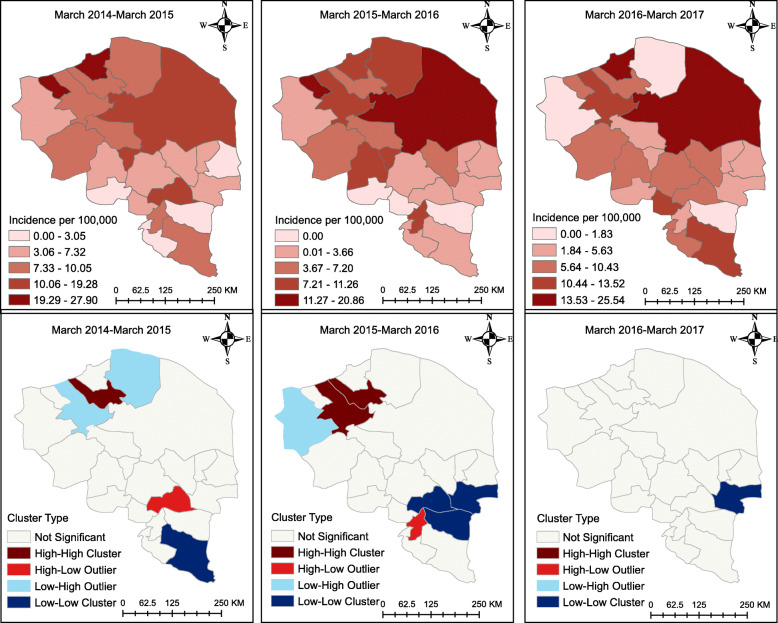
Prostate cancer incidence map at the county level in the province of Kerman, Iran. The figure was created by authors

The descriptive maps of Fig. 6 reveal that the prostate cancer incidence was highest in the North-East. However, as the cluster maps show, there were HH clusters of prostate cancer in the north-western part of the province from 2014 to 2016; furthermore, there were LL clusters of prostate cancer in the South-East and East of the province from 2014 to 2017. All clusters and outliers in the figure were statistically significant (*p* < 0.05).

**Fig. 7:**
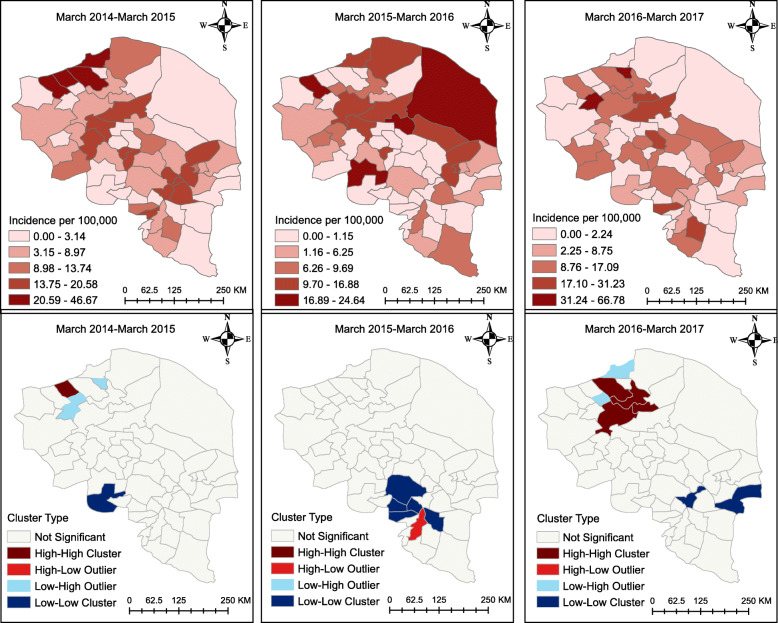
Prostate cancer incidence map at the district level in the province of Kerman, Iran. The figure was created by authors

The descriptive maps of Fig. 7 reveal that the prostate cancer incidence was highest in the North-East of the province from 2015 to 2016. However, there was a HH cluster of prostate cancer in the North-West from 2014 to 2015 and some HH clusters in the North-West stretching towards the centre from 2016 to 2017. All clusters and outliers in the figure were statistically significant (*p* < 0.05).

**Fig. 8:**
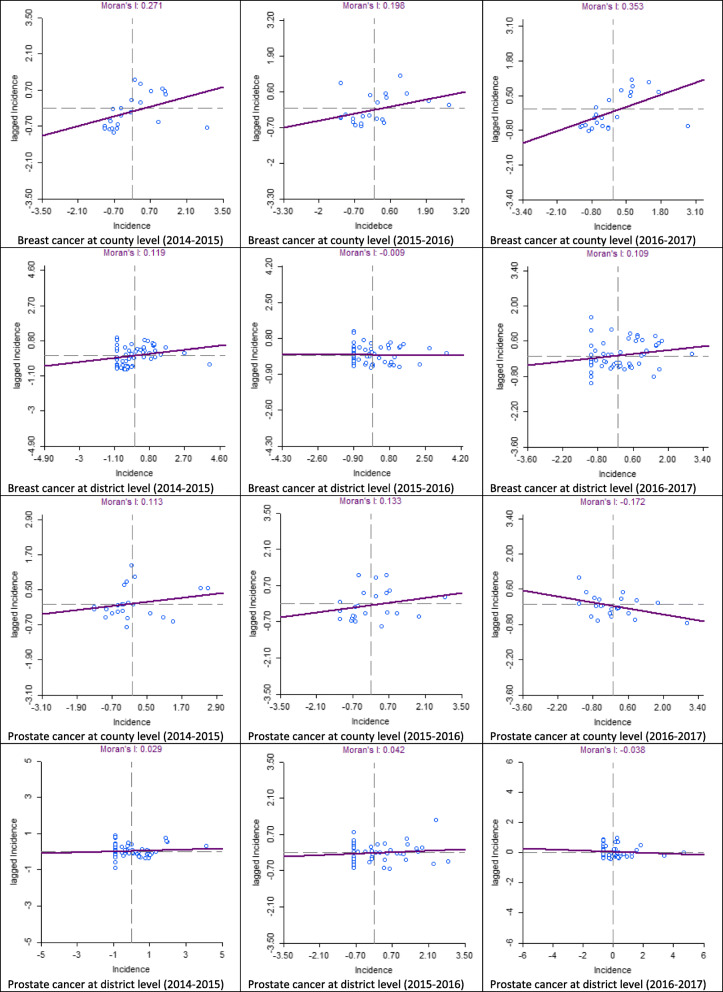
Moran’s scatter plots for breast and prostate cancers cluster maps

The four categories shown in Figs. 4, 5, 6, 7 correspond to the four quadrants in the Moran scatter plots as shown in Fig. 8. If nearby or neighbouring areas are more alike, this is understood as positive spatial autocorrelation. Negative autocorrelation describes patterns in which neighbouring areas are unlike. For example, the first scatter plot (which shows the county level breast cancer for 2014–2015) has a Moran’s value of 0.271 that should be interpreted as a spatial pattern with a cluster tendency (HH and LL). Note that the incidence values have been standardized and are given in standard deviational units (the mean is zero and the standard deviation is 1). Similarly, the spatial lag was computed for those standardized values.

## Discussion

The main aim of this study was to explore the spatio-temporal patterns of the incidence of breast and prostate cancer at a high-resolution geography scale. To the best of our knowledge, this is the first study to assess spatial variations in the incidence pattern of breast and prostate cancers in Iran. The study area in the South of Iran revealed a high incidence rate of both cancers in north-western Kerman, while it was low in the south-eastern part of the province. The number of people with breast or prostate cancer increased considerably after patients reached 25 and 45 years of age, respectively. Further investigations are needed to assess the drivers in the high-risk areas identified in north-western Kerman. They might be associated with environmental factors and lifestyles [12], poor access to cancer-specific services [29], hereditary reasons [10] and/or socio-economic inequalities [30, 31].

Environmental risk factors such as air pollution [32–34] and presence of heavy metals [35, 36] could be linked to the geographic outcome disparities for both cancer forms. We found high-risk areas for the whole study period of breast and prostate cancers in the north-western part of Kerman, an area extending southeast of the Iranian volcano-plutonic copper belt [37] where arsenic contamination is one of the most significant environmental concerns [38]. This is the location of the Sarcheshmeh copper industrial plant, the biggest copper mine of Iran, and it is likely that the neighbourhood is contaminated also by other heavy metals. Field studies report widely distributed travertine rocks north of the Sarcheshmeh copper mine indicating the presence of a highly concentrated range of arsenic compounds [38], which could seep into the water system and contaminate the drinking water in nearby urban and rural communities [38, 39]. Indeed, the arsenic concentration in the water in these areas is higher than the limit recommended by the World Health Organisation (WHO) for drinking water [38, 40, 41]. Arsenic has been categorised as a Group 1 carcinogen factor by the International Agency for Research on Cancer (IARC) [42] and various studies associate arsenic and breast cancer [43–46]; its presence in the study area is thus a potential explanation for the increased incidence of breast cancer found. However, it should also be mentioned that other studies do not show any significant association between breast cancer and arsenic [47, 48]. However, it is conceivable that he power of this association can change due to local and individual diversities [45].

Previous studies indicate a significant association between arsenic-enriched water and prostate cancer incidence [46, 49, 50], while increased levels of copper has been linked with the initiation of prostate cancer [35]. Copper smelting and toxic discharges have led to soil pollution, especially in the region of the smelting plant in Sarcheshmeh Copper Complex. Importantly, the most contaminated areas are located in the most common wind directions [51], and it is particularly disturbing that the polluted areas are also used as grazing land enabling toxic elements from this soil to enter the food chain. These elements include various heavy metals in addition to copper and arsenic, e.g., lead, molybdenum and cadmium [51]. Therefore, soil, water and nutrition of Rafsanjan and the adjacent townships, located in the north-western part of the province are subject to these potential negative effects. Indeed, previous studies have found associations between heavy metals and both breast and prostate cancers [52–54]. The current study strongly recommends examining the hypothesis that exposure to heavy metals, especially arsenic and copper, may be associated with high incidence of gender-specific cancers. In fact, the high incidence of both breast and prostate cancers in north-western Kerman may be associated with these contaminants and this need to be investigated in future studies.

Air pollution, such as that due to particulate matter, has been shown to be associated with breast cancer [33, 34]. Further studies are suggested to confirm the effects of airborne pollution on the risk of breast cancer, especially particulate matter. Fazzo et al. (2016) used a spatial approach to estimate the industrial air pollution impact on 17 selected neoplasms in the territory around the industrial Sicilian area of Priolo, Italy. This area has been defined as a contaminated site of national priority for remediation because of diffuse environmental contamination caused by large industrial settlements, and their study found a higher incidence of breast cancer in the contaminated area compared to the rest of the province [55].

Previous studies highlight that poor access to health care services, such as increased incidence of cancer [29, 56], lead to poor health outcomes [57, 58]. The high incidence of gender-specific cancers in some regions of the study area may be due to their considerable distance from the provincial capital with limited cancer screening programmes. On the other hand, parts of the study area in the South had the lowest incidence of both cancers investigated here although those affected were located even further away from the provincial capital. However, proximity to health care services does not directly translate into access because of potential factors such as poor socio-economic status and low level of education that also are associated with poor access [57]. GIS enable researchers to assess the revealed access to cancer services through combing spatial and non-spatial factors [58–60] and the results suggest measuring access to cancer prevention programmes should be the first step when examining this hypothesis. Previous studies have highlighted the impact of the socio-economic status on the differences in the incidence of cancers of the breast and prostate [61–63]. Assessing the impact of socio-economic status on the geographic disparities of the gender-specific cancers incidence in the study area can be done by analysing the overall spatial structure or identifying high-risk areas. This also warrants further studies.

Hereditary cancer syndromes, a type of inherited disorder in which there is a higher-than-normal risk of certain types of cancer, are caused by mutations in certain genes passed from parents to children [64–68]. Certain such family-related cancers are well-known, e.g., hereditary breast cancer [67] and Lynch syndrome, which is a hereditary non-polyposis colorectal cancer [65]. Hereditary cancer screening programmes [69–71] have made it possible to detect many of the approximately 5–10% of breast cancers caused by a genetic predisposition [72, 73] thus making it possible to prevent them before they occur. There are also studies assessing the risk of prostate cancer associated with hereditary cancer syndromes. This highlights the risk of prostate cancer in members of families associated with early-onset diseases of various kinds [68]. We strongly recommend researchers and policymakers to perform hereditary cancer screening and genetic testing in areas of the province with a high incidence rate of cancer of either breast or prostate.

Spatio-temporal cluster analysis plays a significant role in visualising and quantifying geographical variation in patterns of disease incidence. Global Moran’s *I* and Getis-Ord General G statistic are both global cluster methods which can be used to investigate the level of spatial autocorrelation of disease patterns, while Local Moran’s *I* and Getis-Ord Gi* are indicate the location of the clusters. Although Getis-Ord Gi* statistic is used for identifying hotspots and the opposite, Local Moran’s *I* is also effective for assessing statistically significant spatial outliers [74] and has therefore been predominantly applied [28, 75] and successfully assessed the hotspots [76, 77]. Those using these methods for analysis of the spatial pattern of incident data should consider aggregating the incident data into polygons. The main question here is the geographical scale that should be used for aggregation because it could affect the results. In this study, we conducted the analyses both at the county and the district level, which are geographical scales providing practically useful information. We recommend other researchers performing cohort studies examining the hypotheses proposed here.

### Limitations and future course of the study

We included all patients with breast and prostate cancer in the province of Kerman during 2014–2017. However, we did not have the migration data and used the current address of patients at the time of cancer diagnosis as the patients’ residence. However, some patients might have lived in other regions in the years that might have affected their health.

## Conclusions

We identified a great deal of spatial variations with significant clusters in the patterns of cancer of the breast and the prostate. This suggests that policymakers need to develop prevention strategies tailored to areas where the risk of these conditions are greater than elsewhere. Further, there is a need to conduct further research to test the causal relationship between environmental risk factors and cancer incidence.

## Supplementary Information

**Additional file 1.**

**Additional file 2.**

## Data Availability

The cancers data have been uploaded as supplementary files. However, due to protect the patients’ data, the latitude/longitude of patients’ location has been removed.

## Abbreviations

GIS: Geographical information systems
HH: High-high
LL: Low-low
HL: High-low
LH: Low-high
WHO: World health Organization
IARC: International agency for research on cancer
